# Correlation Between Soluble Klotho and Vascular Calcification in Chronic Kidney Disease: A Meta-Analysis and Systematic Review

**DOI:** 10.3389/fphys.2021.711904

**Published:** 2021-08-13

**Authors:** QiFeng Liu, LiXia Yu, XiaoYa Yin, JianMing Ye, ShaSha Li

**Affiliations:** ^1^Department of Nephrology, Affiliated Kunshan Hospital of Jiangsu University, Kunshan, China; ^2^Clinical Research & Lab Centre, Affiliated Kunshan Hospital of Jiangsu University, Kunshan, China

**Keywords:** sKlotho, vascular calcifcation, chronic kidney diease, biomarker, management

## Abstract

**Background:** The correlation between soluble Klotho (sKlotho) level and vascular calcification (VC) in patients with chronic kidney disease (CKD) remains controversial. Using meta-analysis, we aimed to address this controversy and assess the feasibility of applying sKlotho as a biomarker for VC.

**Methods:** Medical electronic databases were thoroughly searched for eligible publications on the association between sKlotho level and VC in CKD patients. Effectors, including correlation coefficients (*r*), odds ratios (ORs), hazard ratio (HR) or β-values, and 95% confidence intervals (CIs) were extracted and combined according to study design or effector calculation method. Pooled effectors were generated using both random-effects models and fixed-effects models according to *I*^2^-value. Origin of heterogeneity was explored by sensitivity analysis and subgroup analysis.

**Results:** Ten studies with 1,204 participants from a total of 1,199 publications were eligible and included in this meta-analysis. The combined correlation coefficient (*r*) was [−0.33 (−0.62, −0.04)] with significant heterogeneity (*I*^2^ = 89%, *p* < 0.001) based on Spearman correlation analysis, and this significant association was also demonstrated in subgroups. There was no evidence of publication bias. The combined OR was [3.27 (1.70, 6.30)] with no evidence of heterogeneity (*I*^2^ = 0%, *p* = 0.48) when sKlotho was treated as a categorical variable or [1.05 (1.01, 1.09)] with moderate heterogeneity (*I*^2^ = 63%, *p* = 0.10) when sKlotho was treated as a continuous variable based on multivariate logistic regression. No significant association was observed and the pooled OR was [0.29 (0.01, 11.15)] with high heterogeneity (*I*^2^ = 96%, *p* < 0.001) according to multivariate linear regression analysis. There was an inverse association between sKlotho and parathyroid hormone levels. The combined coefficient (*r*) was [−0.20 (−0.40, −0.01)] with significant heterogeneity (*I*^2^ = 86%, *p* < 0.001), and without obvious publication bias. No significant association was found between sKlotho and calcium or phosphate levels.

**Conclusion:** There exists a significant association between decreased sKlotho level and increased risk of VC in CKD patients. This raises the possibility of applying sKlotho as a biomarker for VC in CKD populations. Large, prospective, well-designed studies or interventional clinical trials are required to validate our findings.

## Introduction

Numerous studies have determined that chronic kidney disease (CKD) imparts higher risk of cardiovascular (CV) events and mortality. These detrimental outcomes are caused in part by the presence of a clustering of risk factors such as age, dyslipidemia, aberrant mineral metabolism, comorbidities, and vascular diseases, including vascular calcification (VC) (Baber et al., 2008; Turin et al., 2012; Wan et al., 2020). VC is caused by abnormal mineral deposition in the vascular system (i.e., arterial walls, valves, and the heart) (Chen and Moe, 2015), and is commonly observed in patients with CKD, notably those with end-stage kidney disease (ESKD) (Nakayama et al., 2013; Yao et al., 2017). Importantly, the risk of CV events or mortality in this population is further increased because of the presence of VC, suggesting VC plays a pronounced role in promoting the consequences of kidney disease (Wang Z. et al., 2018). Indeed, it is increasingly believed the presence of VC confers additional CV events and increased mortality risk, and independently predicts more adverse clinical outcomes in CKD populations (Wang Z. et al., 2018; Wang et al., 2019). Therefore, reversing or inhibiting VC may contribute to improved survival. As such, VC is a proposed target for treatment of CKD (Nelson et al., 2020), and screening and establishing surrogate markers of VC is crucial for development of therapeutic agents and improvement of prognosis.

Klotho is a single-chain transmembrane protein (also known as mKlotho) produced primarily by the kidneys (Hu et al., 2016). The extracellular domain of mKlotho can be cleaved by secretases and released into circulation, thereby generating soluble, or secreted Klotho (sKlotho) (Dalton et al., 2017). sKlotho functions as a humoral factor and exerts multiple cytoprotective effects, such as suppression of inflammation, oxidative stress, and fibrosis (Dalton et al., 2017). As a renoprotective protein, sKlotho level is universally downregulated under conditions of CKD (Liu et al., 2017; Wang Q. et al., 2018; Buchanan et al., 2020). Furthermore, loss of sKlotho was shown to be associated with increased inflammation and oxidative stress, and aberrant mineral metabolism, which are risk factors for adverse outcomes (Oh et al., 2015; Almroth et al., 2016; Kawai, 2016). More importantly, sKlotho deficiency promoted VC, whereas sKlotho upregulation or sKlotho therapy significantly ameliorated VC in numerous studies *in vivo and in vitro* (Hu et al., 2011; Hum et al., 2017; Chen et al., 2019). According to these observations, sKlotho is implicated in the process of VC and is increasingly regarded as a novel player and target for intervention against VC (Fukumoto, 2016). Although this notion has been extensively investigated and validated in recent preclinical studies, clinical studies on this association in human CKD populations yielded inconsistent or even contradictory results (Paoli and Mitsnefes, 2014; Cai et al., 2015; Morita et al., 2015; Zheng et al., 2018; Nattero-Chávez et al., 2019; Savvoulidis et al., 2020). The association between sKlotho and VC in the clinical setting remains uncertain and is an area of ongoing study. Therefore, the aim of this meta-analysis was to address these inconsistencies. In this study, we systematically assessed whether decreased sKlotho level was correlated significantly with the presence or degree of VC from the perspective of clinical implication, and we aimed to evaluate the performance of sKlotho as a biomarker for VC detection and management based on evidence from observational or cohort studies in CKD patients.

## Materials and Methods

### Publication Search Strategy

The PubMed, EMBASE, Web of Science, and Cochrane Library electronic databases were thoroughly searched for relevant publications from inception to March 31, 2021. Meta-analysis was performed according to Meta-analysis of Observational Studies in Epidemiology (MOOSE) reporting guidelines (Stroup et al., 2000; Brooke et al., 2021). We employed a PICOM search strategy as follows:

**Patients:** CKD patients undergoing predialysis care or dialysis
**Intervention:** sKlotho level
**Comparison:** Low sKlotho level vs. high sKlotho level
**Outcomes:** Vascular calcification or valvular calcification
**Methods:** Observational or retrospective or prospective cohort study

**Patients:** chronic kidney disease or CKD or chronic renal insufficiency or chronic renal failure or chronic kidney insufficiency or chronic nephropathy or chronic renal disease or chronic kidney failure or end stage renal disease or end stage kidney disease or ESRD or ESKD or pre-dialysis or dialysis or renal dialysis or uremic or uremia or hemodialysis or HD or peritoneal dialysis or PD.

**Intervention:** Klotho or soluble Klotho or secreted Klotho or sKlotho or alpha-Klotho or α-Klotho or KL or sKL or αKL.

**Comparison:** correlation between sKlotho level and VC.

**Outcomes:** calcification or vascular calcification or blood vessel calcification or arterial calcification or aorta calcification or coronary artery calcification or valve calcification or heart calcification or calcified blood vessel or calcified vasculature or calcified artery or calcified valve.

An additional search of references from potential articles on this topic was performed manually.

Additionally, associations between sKlotho and non-VC clinical outcomes in the included studies were recorded and investigated.

### Inclusion and Exclusion Criteria

Eligible studies were required to meet the following inclusion criteria: (1) adult participants (age ≥ 18 years); (2) observational or cohort study design; (3) explored whether sKlotho level was associated with VC in CKD patients undergoing predialysis care or dialysis; and (4) published in English.

Exclusion criteria were as follows: (1) studies with incomplete or insufficient data; (2) duplicated studies; and (3) lack of human or relevant studies (i.e., studies on the association between mKlotho or urinary sKlotho and VC were excluded, as were case reports and reviews).

### Data Extraction

Data extraction was performed by two independent investigators (QiFeng Liu and XiaoYa Yin) using a standardized form. Extracted data were as follows: first author, publication year, country, sample size, age, research methodology, follow-up duration, correlation coefficient (*r*), β-value, odds ratio (OR), relative risk (RR), or hazard ratio (HR) with 95% confidence intervals (CIs). Reported Pearson correlation coefficients (*r*) were affected by logarithmic transformation; therefore, they were converted to Spearman correlation coefficients (*r*) as previously described (Tierney et al., 2007; Chen et al., 2013; Liu et al., 2019). β-values were converted to ORs using exp(β). If effectors including *r*, β-value, and OR with corresponding CIs were not extracted directly from a report, the corresponding author was contacted for the relevant data. Otherwise, indirect effectors with CIs were calculated according to the raw data presented in the original article. Any disagreements were resolved by discussion to achieve consensus. Study quality and bias risk were assessed by two authors (LiXia Yu and JianMing Ye) using criteria of the Newcastle-Ottawa Scale (NOS) for case-control or cohort studies. High quality studies were defined as studies with NOS score >7.0.

### Statistical Analysis

Each Spearman correlation coefficient (r) was converted to a *Z*-value via Fisher's transformation, which was approximately normally distributed. Standard error (SE) of *Z* was calculated and *Z*-values were converted via inverse Fisher's transformation to generate *r* and 95% CI, which were then combined to produce summary *r* values and 95%CIs. Extracted ORs, HRs, or exp(β) with 95% CIs were, respectively, pooled to generate the overall effect size of the association between sKlotho and VC (including valvular calcification) using Review Manager 5.3 (Cochrane Collaboration, Copenhagen, Denmark). Heterogeneity across studies was determined using the chi-squared test and *I*^2^ statistics. A fixed-effects model was used to summarize the pooled effectors if *I*^2^ ≤ 50%. Otherwise, a random-effects model was used. Sensitivity analysis was performed to guarantee consistency of the results by omitting individual studies one by one. If substantial heterogeneity occurred, subgroup analysis was employed to find the sources of heterogeneity. Additionally, the possibility of publication bias was examined visually using a funnel plot and further tested using Begg's and Egger's tests using Stata12.0 (StataCorp LP, College Station, TX, USA). Statistical significance was set at two-tailed *p* < 0.05.

## Results

### Search Results and Study Selection

**Figure 1:**
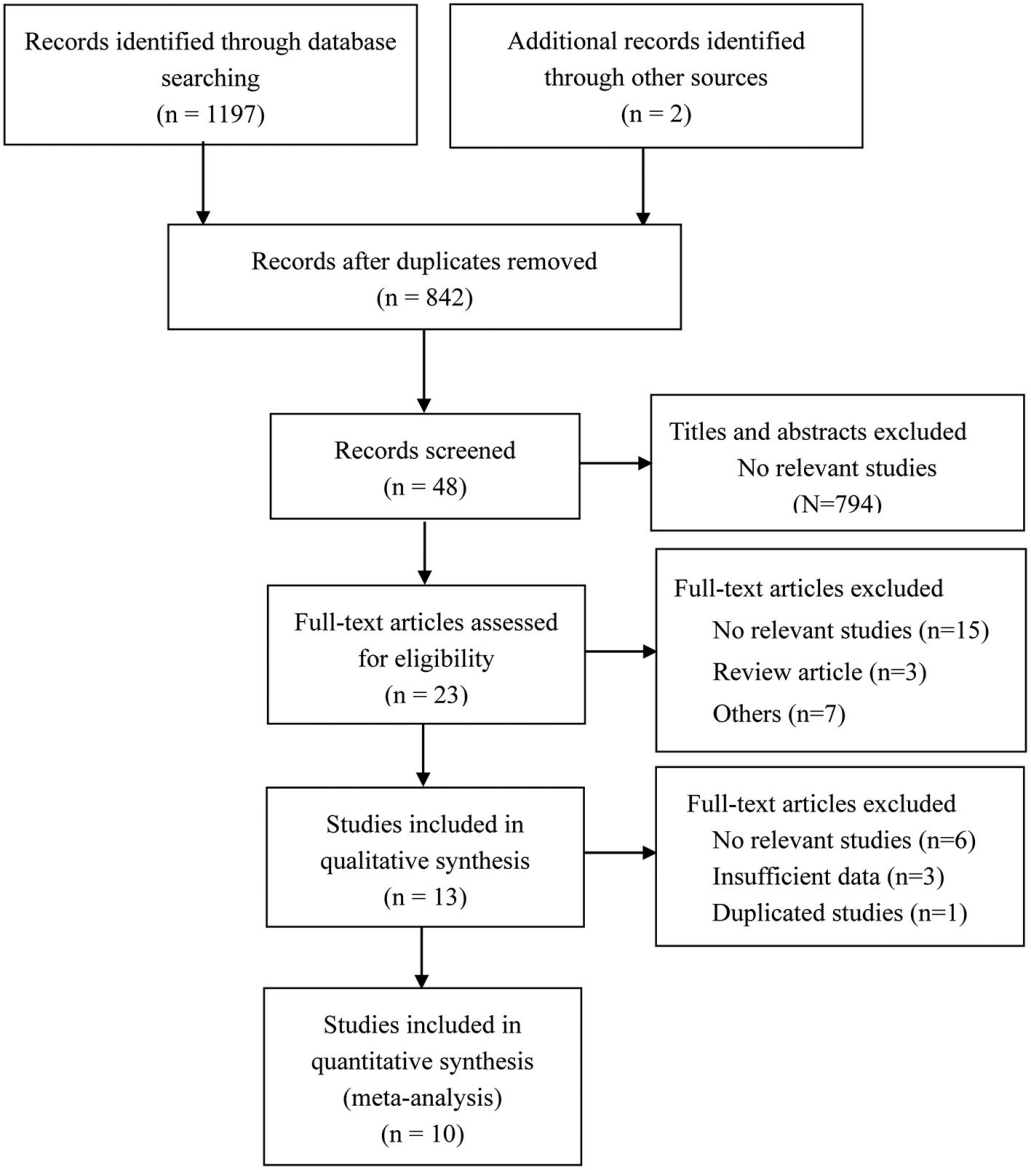
Flow chart of the included studies in the meta-analysis.

The Preferred Reporting Items for Systematic Reviews and Meta-Analyses (PRISMA) diagram of the study selection process is shown in Figure 1. Our systematic search yielded 1,197 articles, and search of references yielded two additional articles. A total of 357 articles were removed after deduplication, with 842 remaining. Next, 794 were discarded after reviewing titles and abstracts, with 48 remaining. An additional 25 were excluded after full-text assessment, with 23 remaining. A further 10 were precluded after thoroughly reading the full text of the remaining 23 articles. Ultimately, 10 articles were included in our meta-analysis.

### Study Features

Ten publications, involving 1,204 participants were eligible according to the inclusion and exclusion criteria (Kitagawa et al., 2013; Buiten et al., 2014; Cai et al., 2015; Di Lullo et al., 2015; Morita et al., 2015; Krishnasamy et al., 2017; Zheng et al., 2018; Koga et al., 2020; Xu et al., 2020; Chen et al., 2021). All included studies had observational or cross-sectional design. Primary features of the included studies and baseline information on participants are shown in Table 1. Among these studies, five reported correlation coefficients (*r*) pertaining to the association between sKlotho level and VC using Pearson correlation or Spearman correlation analysis (Cai et al., 2015; Di Lullo et al., 2015; Krishnasamy et al., 2017; Zheng et al., 2018; Koga et al., 2020); five reported ORs and CIs pertaining to the association between sKlotho level and VC according to multivariate logistic regression analysis (Cai et al., 2015; Morita et al., 2015; Zheng et al., 2018; Koga et al., 2020; Chen et al., 2021); one study only reported a β-value in multivariate logistic regression analysis (Kitagawa et al., 2013); and two studies reported β-values and CIs pertaining to the association between sKlotho level and VC according to multivariate linear regression analysis (Buiten et al., 2014; Xu et al., 2020). Regarding the correlation between sKlotho and non-VC clinical findings among the included studies, one study reported ORs and CIs pertaining to the association between sKlotho level and vascular dysfunction (Kitagawa et al., 2013) or cardiovascular events (Buiten et al., 2014) according to multivariate logistic regression analysis. One study reported HRs and CIs pertaining to the association between sKlotho level and all-cause mortality according to Cox regression analysis (Zheng et al., 2018). Another study reported β-values and CIs pertaining to the association between sKlotho level and carotid intima-media thickness according to multivariate linear regression analysis (Xu et al., 2020). However, no studies reported results on the association between VC and clinical outcomes. Consequently, the effectors were, respectively, combined based on specific data presentation or study design (such as correlation analysis or multiple regression analysis). According to the NOS for case-control studies, the quality of the 10 included studies was assessed. Five were graded as good, two as fair, and three as low. The average score was 7.0 and details are shown in Table 2.

**Table 1:** Characteristics of the included studies.

**References**	**Country**	**Study design**	***N***	**Average age**	**Disease** **models**	**Outcomes**	**Correlation r**	**Effectors and CIs**	**Relationship**
Chen et al. (2021)	China	Cross-sectional	180	58 ± 14	CKD2-5	Valve calcification	No data	OR: 1.075 (1.027–1.126)	Inverse
Xu et al. (2020)	China	Cross-sectional	112	60.13 ± 9.30	MHD	AAC	No data	β: −3.128 (−4.499 to −1.758)	Inverse
			Before-after						
Koga et al. (2020)	Japan	Cross-sectional	75	68 ± 9	CKD1-4	CAC	−0.032, *p* = 0.006★	OR: 6.94 (1.26–38.24)	Inverse
								No data	
Zheng et al. (2018)	China	Cross-sectional	128	61.91 ± 15.39	MHD	CAC	−0.667, *p* = 0.001★	OR: 1.033 (1.020–1.044)	Inverse
			Prospective						
Krishnasamy et al. (2017)	Australia	Prospective	82	62.9 ± 10.2	CKD4-5 42 controls	AAC	−0.36, *p* = 0.002★	No data	No
Di Lullo et al. (2015)	Italy	Cross-sectional	100	51 (46–56)	CKD3-4	Valve calcification	−0.208; *p* = 0.04	No data	No
Cai et al. (2015)	China	Cross-sectional	129	58.18 ± 13.72	MHD	AAC	−0.214, *p* = 0.015	OR:3.559 (1.453–8.717)	Inverse
Morita et al. (2015)	Japan	Cross-sectional	157	W:65.8 ± 11.5	CKD2	CAC	No data	OR: CAC 2.0 (0.625–6.25)	No
				M:67.0 ± 11.6	CKD2	AVC	No data	OR: AVC 0.34 (0.11–1.02)	No
Buiten et al. (2014)	UK	Cross-sectional	127	67 ± 7	MHD	AAC	No data	β*:* 0.58 (−0.07–1.22)	No
						CAC	No data	β: 0.08 (−0.19–0.36)	No
Kitagawa et al. (2013)	Japan	Cross-sectional	114	58 (47–66)	CKD1-3	ACI	No data	β: −0.00226, *p* = 0.251	No

★ *, Spearman relation; CKD, chronic kidney disease; MHD, maintenance hemodialysis; OR, odds ration; CI, confidence interval; ESKD, end stage kidney disease; W, women; M, men; MAC, medial arterial calcification; CAC, coronary artery calcification; AVC, aortic valve calcification; AAC, abdominal aorta calcification; ACI, aortic calcification index*.

**Table 2:** NOS scores of the case-control studies included.

**Case-control study**	**Is the case definition adequate?**	**Representativeness of the cases**	**Selection of Controls**	**Definition of Controls**	Control for important factor^*^	Control for additional factor^*^	Ascertainment of exposure^*^	**Same method of ascertainment for cases and controls**	**Non-Response rate**	**Total quality scores**
Chen et al. (2021)	★	★	★	★	★	★	★	★	/	8
Xu et al. (2020)	★	★	★	★	★	/	/	★	/	6
Koga et al. (2020)	★	★	★	★	★	★	★	★	/	7
Zheng et al. (2018)	★	★	★	★	★	★	/	★	/	8
Krishnasamy et al. (2017)	★	★	★	★	★	★	★	★	/	8
Di Lullo et al. (2015)	★	★	/	/	★	★	★	/	/	5
Cai et al. (2015)	★	★	★	★	★	★	★	★	/	8
Morita et al. (2015)	★	/	★	★	★	★	★	★	/	7
Buiten et al. (2014)	★	★	★	★	★	★	★	★	/	8
Kitagawa et al. (2013)	★	★	/	/	★	★	★	/	/	5

* *2 stars could be awarded for this item. Studies that controlled for age or renal function or indicators of calcium and phosphorus metabolism was awarded one star, respectively*.

*NOS, Newcastle–Ottawa Scale*.

### The Association Between sKlotho and VC According to Spearman Correlation

**Figure 2:**
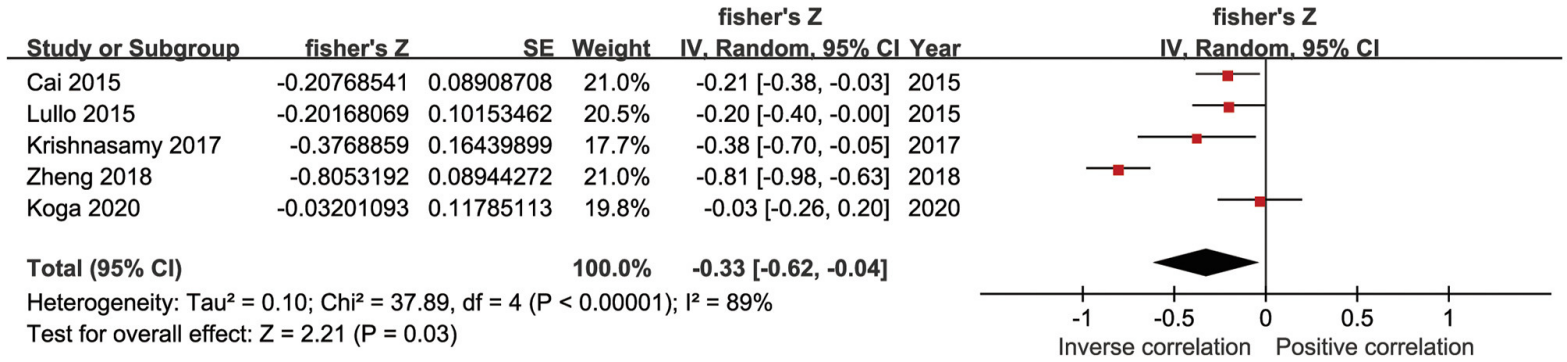
Forest plots of the summary r for the association between sKlotho level and VC.

**Figure 3:**
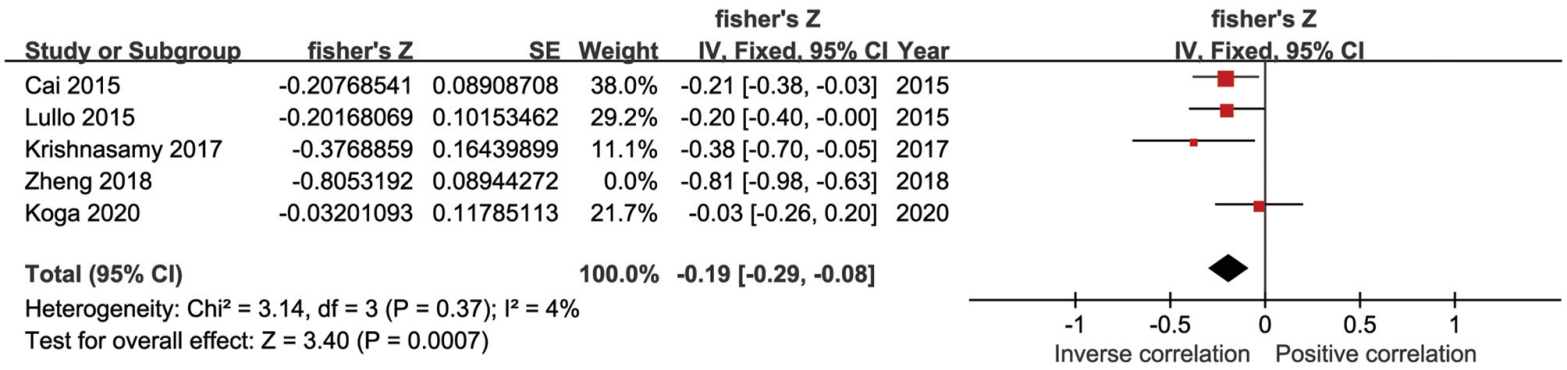
Forest plots of the summary r after removing Zheng's study.

**Figure 4:**
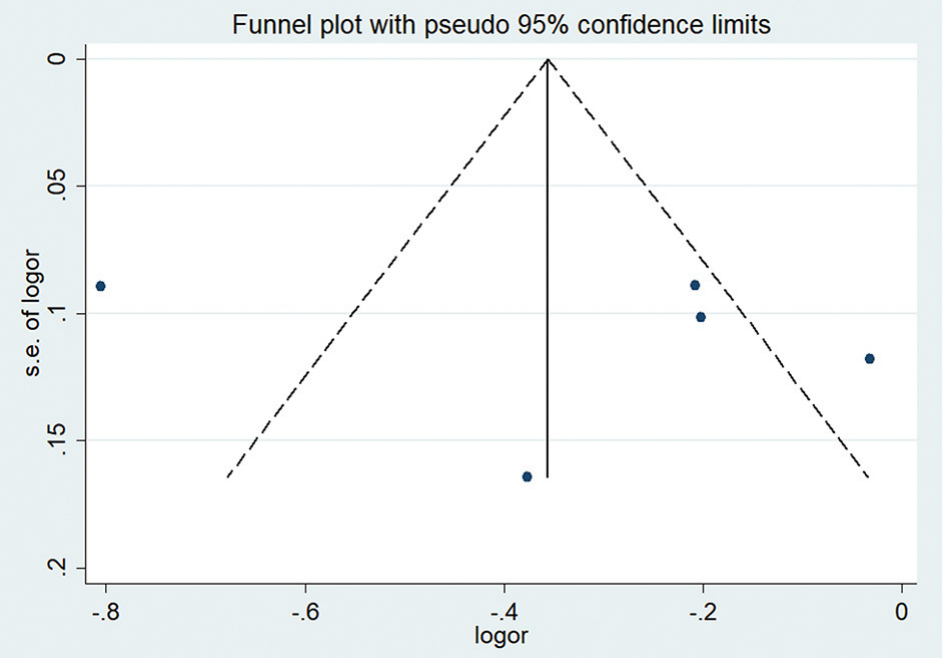
Funnel plot of sKlotho levels and VC.

In five studies, there was an inverse association between sKlotho level and VC according to Spearman or Pearson correlation (*r*) analysis. VC was assessed as either abdominal aorta calcification (AAC) (Cai et al., 2015; Krishnasamy et al., 2017), coronary artery calcification (CAC) (Zheng et al., 2018; Koga et al., 2020), or valvular calcification (Di Lullo et al., 2015). Because of substantial heterogeneity among these five studies (*I*^2^ = 89%, *p* < 0.001), a random-effects model was used for data synthesis. The combined correlation coefficient(*r*) was [−0.33 (−0.62, −0.04)], which suggested a significant negative association between sKlotho level and VC (Figure 2). Subgroup analysis was also performed to search the potential causes of high heterogeneity according to age, sample size, disease models, location of VC, and study quality. Although similar associations were observed, significant heterogeneity existed in all subgroups (Table 3). Notably, significant heterogeneity disappeared in sensitivity analysis (*I*^2^ = 4%, *p* = 0.37), while the combined result persisted [−0.19 (−0.29, −0.08)] after removing the study by Zheng et al. (Figure 3), suggesting this study had a profound effect on heterogeneity and may have been the source of high heterogeneity. No apparent publication bias was observed according to Begg's test (*p* = 1.0) or Egger's test (*p* = 0.647) (Figure 4).

**Table 3:** Results of subgroup analysis by age, sample size, disease models, location of VC and study quality.

**Subgroup**	**Studies**	**Effect estimate (Random-effects model)** **Pooled r [95% CI]**	**heterogeneity** **Between subgroup**
Age	5		
Age ≥ 60 years	3	−0.41 [−0.92, 0.10]	*P* < 0.001; *I^2^* = 93%
Age <60 years	2	−0.21 [−0.34, −0.07]	*p* = 0.96; *I^2^* = 0%
Sample size	5		
Sample size ≥ 100	3	−0.41 [−0.81, −0.01]	*P* < 0.001; *I^2^* = 93%
Sample size <100	2	−0.19 [−0.52, 0.15]	*p* = 0.09; *I^2^* = 66%
Disease models	5		
Pre-dialysis	3	−0.18 [−0.35, −0.01]	*p* = 0.22; *I^2^* = 34%
Dialysis	2	−0.51 [−1.09, 0.08]	*P* < 0.001; *I^2^* = 96%
Location of VC	5		
AAC	2	−0.25 [−0.40, −0.09]	*p* = 0.37; *I^2^* = 0%
CAC	3	−0.35 [−0.83, 0.13]	*P* < 0.001; *I^2^* = 94%
Study quality	5		
High quality study (> 7 stars)	3	−0.47 [−0.88, −0.05]	*P* < 0.001; *I^2^* = 91%
Low quality study (≤ 7 stars)	2	−0.13 [−0.29, 0.04]	*p* = 0.28; *I^2^* = 16%

### The Association Between sKlotho and VC According to Logistic or Linear Regression

**Figure 5:**
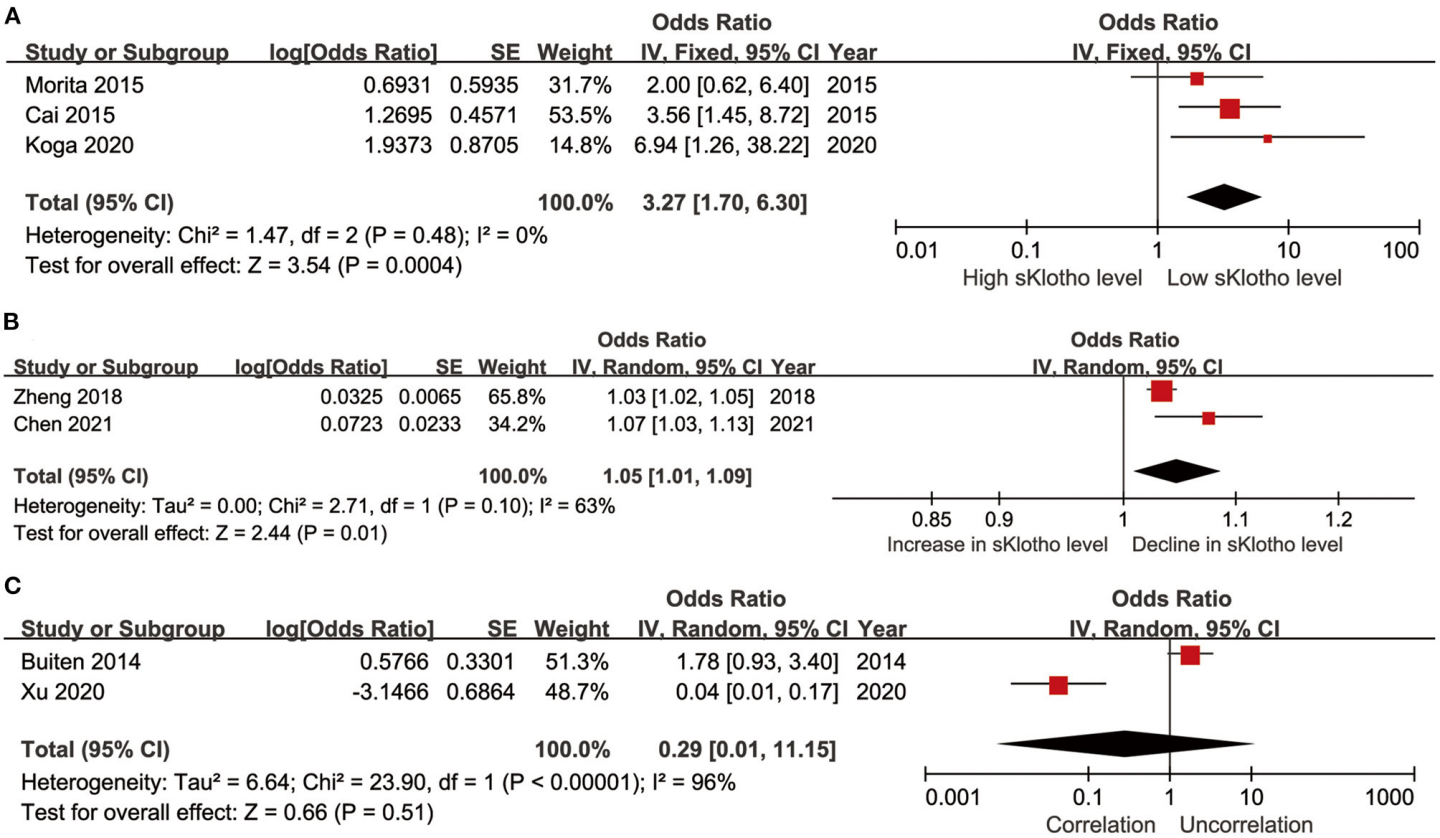
Forest plots of the summary OR for the association between sKlotho level and VC; **(A)** sKlotho as a categorical variable; **(B)** sKlotho as a continuous variable; **(C)** Result of multivariate linear regression analysis.

Five studies reported ORs and CIs generated using multivariate logistic regression (Cai et al., 2015; Morita et al., 2015; Zheng et al., 2018; Koga et al., 2020; Chen et al., 2021). The extracted effectors in the original studies were generated after adjustment for important confounders including age, mineral metabolism, kidney function, and comorbidities. In these studies, sKlotho was treated either as a categorical variable (sKlotho tertiles or quartiles or median sKlotho value) (Cai et al., 2015; Morita et al., 2015; Koga et al., 2020) or continuous variable (overall sKlotho level) in regression models (Zheng et al., 2018; Chen et al., 2021). Therefore, we, respectively, calculated the combined effectors based on treatment as a categorical or continuous variable. One study reported a β-value, although a corresponding CI was not provided (Kitagawa et al., 2013). This study was therefore not combined for meta-analysis. The pooled effector OR was [3.27 (1.70, 6.30)] with no heterogeneity (I^2^ = 0%, *p* = 0.48) (Figure 5A) and [1.05 (1.01, 1.09)] with moderate heterogeneity (*I*^2^ = 63%, *p* = 0.10) (Figure 5B) according to whether sKlotho was treated as a categorical or continuous variable, respectively. Two studies reported β-values pertaining to the association between sKlotho level and VC using multivariate linear regression analysis after adjusting for similar confounders (Buiten et al., 2014; Xu et al., 2020). Indirect ORs and CIs were calculated using exp(β). The pooled effector was [0.29 (0.01, 11.15)] with highest heterogeneity (*I*^2^ = 96%, *p* < 0.001) (Figure 5C). No related subgroup analysis was performed because of the limited number of included studies. These results suggest low sKlotho level was correlated closely with high risk of VC according to multivariate logistic regression (except for linear regression analysis).

Regarding the correlation between sKlotho and other non-VC clinical findings, one study reported that low sKlotho level appeared to correlate more with cardiovascular diseases, although this association disappeared [0.91 (0.66–1.23)] in adjusted models (Buiten et al., 2014). One study reported that sKlotho level was not independently associated with all-cause mortality in CKD patients, and the effector was not presented or calculated in the original report (Zheng et al., 2018). According to another study, each 100-pg/mL increase in sKlotho level was associated with a 40% reduction in risk of arterial stiffness (Kitagawa et al., 2013). Interestingly, Xu et al. reported that treatment with tanshinone IIA effectively decreased cardiovascular events in CKD patients, and the beneficial effect exerted by tanshinone IIA was ascribed to the elevation in sKlotho level (Xu et al., 2020). Because of significant differences in nature among these studies, they were unable to be combined to generate pooled effectors.

### The Association Between sKlotho and Calcium, Phosphate, or Intact Parathyroid Hormone

**Figure 6:**
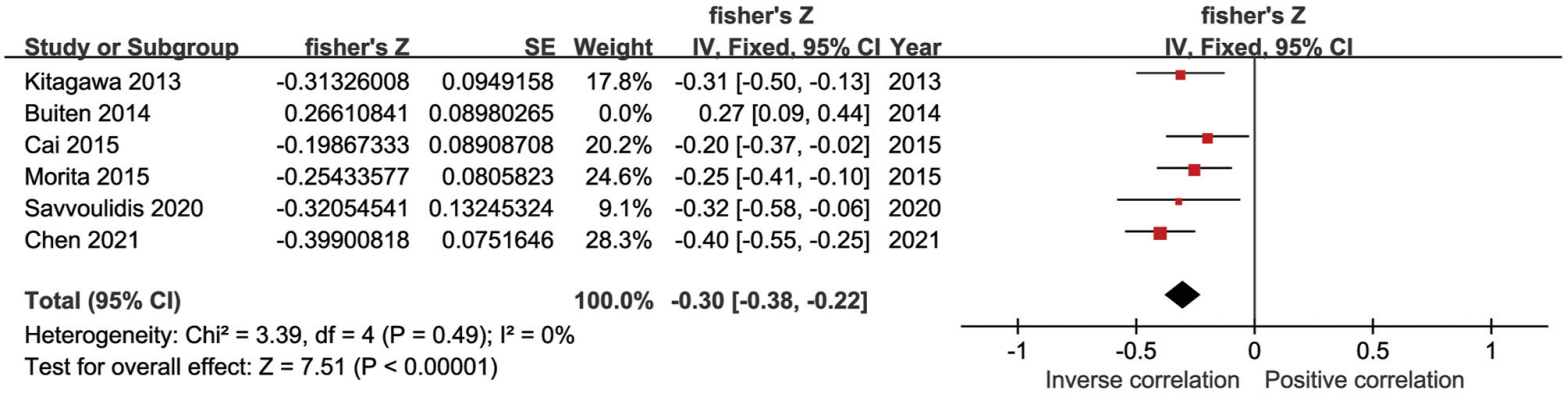
Forest plots of the summary r for the association between sKlotho level and PTH after removing Buiten's study.

**Figure 7:**
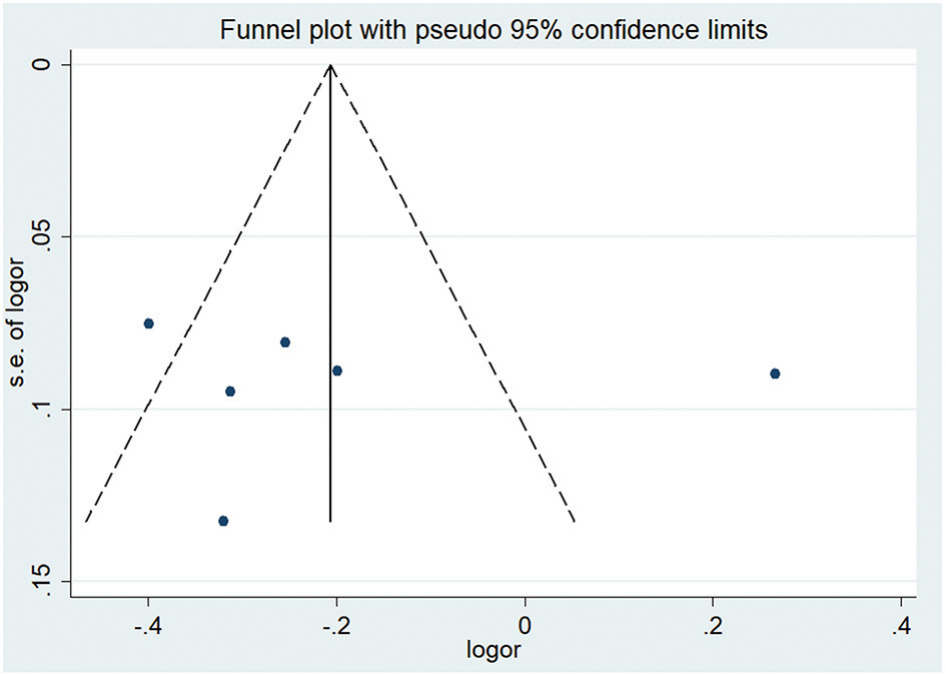
Funnel plot of sKlotho levels and PTH.

Four studies reported correlation coefficients (*r*) pertaining to the association between sKlotho and calcium. Among them, two found a positive association (Cai et al., 2015; Chen et al., 2021), another found a negative association (Buiten et al., 2014), and one found no association (Kitagawa et al., 2013). The pooled coefficient (r) was [0.23 (−0.05, 0.51)] with substantial heterogeneity (*I*^2^ = 91%, *p* < 0.001) (Table 4). Regarding sKlotho and phosphate, four studies found an inverse association (Kitagawa et al., 2013; Buiten et al., 2014; Savvoulidis et al., 2020; Chen et al., 2021) and one study found a positive association (Morita et al., 2015). The pooled coefficient (r) was [−0.17 (−0.42, 0.07)], although with high heterogeneity (*I*^2^ = 89%, *p* < 0.001) (Table 4). Regarding sKlotho and intact parathyroid hormone (PTH), five studies found an inverse association (Kitagawa et al., 2013; Cai et al., 2015; Morita et al., 2015; Savvoulidis et al., 2020; Chen et al., 2021), one found a positive association (Buiten et al., 2014), and the pooled coefficient (*r*) was [−0.20 (−0.40, −0.01)], although again with apparent heterogeneity (*I*^2^ = 86%, *p* < 0.001) (Table 4). Sensitivity analysis revealed that the study by Buiten et al. had a significant impact on heterogeneity (*I*^2^ = 0%, *p* = 0.49) and the overall result was not altered by removal of this study [−0.30 (−0.38, −0.22)] (Figure 6). No obvious publication bias was found with Begg's test (*p* = 0.707) or Egger's test (*p* = 0.857) (Figure 7).

**Table 4:** Results of the association of sKlotho level with calcium, phosphate and PTH.

**Indicators**	**Studies**	**Pooled r [95% CI]**	***P*** **-value**	**Effects model**	**Heterogeneity**
Calcium	4	0.23 [−0.05, 0.51]	0.11	Random–effects model	*P* < 0.001; *I^2^* = 91%
Phosphate	5	−0.17 [−0.42, 0.07]	0.16	Random–effects model	*P* < 0.001; *I^2^* = 89%
PTH	6	−0.20 [−0.40, −0.01]	0.04	Random–effects model	*P* < 0.001; *I^2^* = 86%

*PTH, parathyroid hormone*.

## Discussion

To our knowledge, this is the first study that systematically investigates the association between sKlotho level and VC in CKD patients using meta-analysis. Our findings demonstrate that decreased sKlotho level is associated with increased risk of VC based on Spearman correlation and adjusted logistic regression analyses. To some degree, these findings support the application of sKlotho as a biomarker for management of VC in CKD despite moderate heterogeneity.

VC is known to increase risk of CV morbidity and mortality in CKD patients. Owing to the lack of an ideal biomarker for VC, therapies that effectively reverse or ameliorate VC still not become available in clinics. Klotho was originally identified in 1997 as an aging modulator. In addition to suppression of aging, Klotho exerts substantial protective effects against renal injury resulting from oxidative stress, ischemia, and inflammation. Because Klotho is produced primarily by the kidneys, Klotho deficiency is believed to be a consequence of CKD. Moreover, there is ample evidence that Klotho deficiency worsens kidney function because of the abolition of its renoprotective effects. Klotho deficiency is therefore involved in the development of CKD. VC is frequently observed in CKD, and the prevalence of VC increases with progression of CKD (London et al., 2016). VC is a feature of normal aging and CKD is the state of premature aging (Glassock et al., 2017). Consequently, it is reasonable to postulate that sKlotho deficiency is critical for onset and progression of VC in CKD. Consistent with this notion, systemic knockdown of Klotho in mice resulted in severe VC and ectopic calcification in various organs, whereas overexpression of Klotho in transgenic mice resulted in no or minimal VC and ectopic calcification (Hu et al., 2011). Klotho is also detectable in local vascular tissues, and downregulated vascular Klotho expression was found in early CKD, which promoted VC (Fang et al., 2014). That is, systemic or local Klotho deficiency stimulates the process of VC. The observation that recombinant sKlotho protein or endogenous sKlotho upregulation inhibited VC revealed that sKlotho exerts potential therapeutic effects against VC, and thus it was proposed as a novel target for management of VC (Lau et al., 2012; Lim et al., 2012; Cheng et al., 2017).

The mechanism underlying how sKlotho inhibits VC is complex and multifactorial. VC is defined as mineral deposition in the vascular system. It is derived from osteogenic differentiation of vascular smooth muscle cells (VSMCs), which is driven by runt-related transcription factor 2 (Runt2), similar to bone formation (Qian et al., 2021). Klotho therapy directly downregulated phosphate transport (Pit) 1 and Pit 2 levels, and inhibited Runt2-driven VSMC and valve interstitial cell differentiation (Hu et al., 2011; The et al., 2020). Wnt/β-catenin signaling plays a critical role in development of VC (Rong et al., 2014). Klotho suppressed calcification stimulated by β-glycerophosphate via blocking the bound of β-catenin to the promoter of Runt2 gene in VSMCs (Chen et al., 2015, 2019). Endoplasmic reticulum (ER) stress is activated in the context of CKD, and contributes to VC (Miyazaki-Anzai et al., 2014). We previously reported that Klotho ameliorated ER stress, and this may contribute to the anti-VC effect of Klotho (Liu et al., 2015). Mammalian target of rapamycin (mTOR) signaling was enhanced in calcified aortic wall, and rapamycin administration suppressed VC. However, the ameliorating effect of rapamycin on VC was abolished in the absence of sKlotho. This suggested the beneficial effect of Klotho may be attributed to modulation of mTOR signaling (Zhao et al., 2015). High PTH level is an established risk factor for VC (Chou et al., 2019; Ho et al., 2019). Klotho was found in parathyroid tissues, and suppressed PTH synthesis via a fibroblastic growth factor-23 (FGF23)-dependent mechanism (Ben-Dov et al., 2007). Decreased Klotho expression in CKD caused parathyroid glands to be resistant to FGF23, which resulted in overexpression of PTH (Yan et al., 2015). Accordingly, in this study, we found there indeed exists a negative association between sKlotho and PTH, and means that sKlotho has an inhibitory effect on PTH production. Furthermore, Klotho reduces serum phosphate by suppressing sodium/phosphate cotransporter expression (Ide et al., 2018), and increases urinary calcium reabsorption by affecting renal calcium transport in the kidney (Wolf et al., 2014). Via these mechanisms, Klotho reverses abnormal mineral metabolism, and this may partially lead to amelioration of VC. Therefore, sKlotho appears to be positively associated with calcium and inversely correlated with phosphate. However, these associations did not reach statistical significance in our analyses. Because the correlation coefficients (r) were derived from unadjusted models, the effects of other factors, including FGF23, diet, active vitamin D, and medication on mineral metabolism cannot be fully excluded. Finally, sKlotho suppresses oxidative stress and inflammation in CKD, which are risk factors that promote VC. This may represent another mechanism that contributes to the anti-VC effect of Klotho (Kendrick and Chonchol, 2011; Zeng et al., 2019; Lee et al., 2020). Above all, it was proven that Klotho is implicated in the pathogenesis and progression of VC, which in turn raised the possibility of applying Klotho as a potential biomarker and target for management of VC in CKD.

Increasing attention has been paid to this area, and a growing number of studies have been conducted to investigate whether sKlotho level was associated with VC in clinical practice, although they yielded uncertain results. A previous study of 129 patients on hemodialysis showed that each standard deviation increase in sKlotho level was followed by a 37.1% decrease in AAC score, and the authors proposed that decreased sKlotho level is an independent risk factor for AAC (Cai et al., 2015). A recent study of 180 predialysis patients showed that patients with cardiac valve calcification (CVC) had lower sKlotho level, and low sKlotho level was independently associated with increased CVC (Chen et al., 2021). Similarly, other studies demonstrated an inverse association between sKlotho level and CAC (Zheng et al., 2018; Koga et al., 2020). These observations support clinical application of sKlotho as a biomarker for VC. However, several other studies reported inconsistent findings. In these studies, no apparent association was found between decreased sKlotho level and increased VC (including AAC, CVC, or CAC) in CKD populations using fully adjusted models (Kitagawa et al., 2013; Buiten et al., 2014; van Venrooij et al., 2014; Morita et al., 2015; Krishnasamy et al., 2017). The inconsistent results from clinical studies indicate that the correlation between sKlotho and VC remains to be fully determined.

There are several plausible explanations for the above discrepancies. First, standardization of sKlotho assays has not been achieved. There are commercially available enzyme-linked immunosorbent assays (ELISA) for measuring sKlotho levels. These assays differ significantly in quality with different sensitivities and specificities (Heijboer et al., 2013; Neyra et al., 2020). It is therefore difficult to produce accurate results among assays. A novel sKlotho assay using immunoprecipitation or immunoblot rather than ELISA displays higher performance, and may resolve these inconsistencies in the future (Neyra et al., 2020). Second, whether Klotho is locally expressed in vasculature remains controversial. Several studies suggest Klotho is expressed in calcified vascular tissues (Lim et al., 2012; Ritter et al., 2015), whereas other data showed that Klotho is undetectable or not increased in calcified vascular tissues (van Venrooij et al., 2014; Mencke et al., 2015; Rukov et al., 2016). sKlotho does not necessarily reflect tissue mKlotho levels (Drüeke and Massy, 2013; Olauson et al., 2017). This implies the effects of vascular Klotho or systemic Klotho on VC remain to be clarified. This discrepancy may also be attributable to differences in antibodies against Klotho isoforms (Lewin and Olgaard, 2015). Third, VC progresses slowly in patients with early stages of CKD, compared with those with advanced CKD or ESKD with more pro-calcific risk factors (Krishnasamy et al., 2017; Chen et al., 2021). Differences in disease models potentially contributed to this inconsistency. Fourth, sKlotho level is modulated by many factors including age, oxidative stress, and inflammation (Inci et al., 2016; Ruiz-Andres et al., 2016). Furthermore, sKlotho level is influenced by commonly used agents in CKD, such as phosphate binders, active vitamin D, and renin-angiotensin system inhibitors (Forster et al., 2011; Lin et al., 2014; Inci et al., 2016; Eltablawy et al., 2018; Golmohamadi et al., 2018). Hence, sKlotho level may change over time, and a single measurement at baseline in these studies may not necessarily reflect actual levels. To some degree, these confounders weaken the association between sKlotho and VC. Importantly, to date, there are few related prospective cohort or longitudinal studies available. Reliable conclusions cannot be drawn based on the results of observational or cross-sectional studies because of deficiencies inherent in their nature.

These uncertainties do not necessarily preclude sKlotho from having an effect on VC. Rather, this suggests the involvement of a more complex mechanism underlying the effects of Klotho during development of VC. To address the inconsistencies discussed, we performed the present meta-analysis and systematic review to assess the role of sKlotho in the pathogenesis of VC in CKD patients. We included 10 publications with 1,204 predialysis and dialysis patients. We found there was a significant association between decreased sKlotho level and elevated risk of VC (including AAC, CAC, and valvular calcification). Similar results were also obtained in subgroups when the different coefficients were analyzed. The strong association was further validated following adjustment for potential confounders in multivariate logistic regression. We also found that sKlotho was inversely correlated with risk factors for VC, such as PTH level. These results suggest sKlotho deficiency correlates strongly with increased VC risk, and that sKlotho has the potential to be applied as a marker for detection and evaluation of VC. Notably, we observed that apparent heterogeneity appeared in terms of study designs or methodologies among the 10 eligible studies. To minimize potential heterogeneity among studies, we combined the studies based on specific study design or methodology. From this perspective, the conclusion obtained should be considered relatively reliable, and the finding supports the potential application of sKlotho as a biomarker for management of VC in CKD. This statistically significant association was not demonstrated with linear regression in two of the included studies. However, highest heterogeneity occurred, indicating that these two studies were unsuitable for pooling because of differences in nature.

In the present study, we also investigated the association between sKlotho and non-VC clinical outcomes including vascular function, and cardiovascular morbidity or mortality. There remain controversies regarding the association between sKlotho and cardiovascular morbidity or mortality (Buiten et al., 2014; Zheng et al., 2018). Because of the limited number of eligible studies and substantial heterogeneities among studies, sKlotho is not precluded from being associated with other adverse clinical outcomes. Rather, increasing evidence has demonstrated this significant association and validated the prognostic value of sKlotho in CKD populations (Liu et al., 2020; Yang et al., 2020; Yu et al., 2020).

The present study had several limitations that must be addressed. Firstly, the included studies were observational in design, with selection bias and uncontrolled confounders, and the total number of participants enrolled was relatively small, which only revealed an association. Therefore, the strength of our conclusion may be attenuated and the notion that decreased sKlotho level predicts VC cannot be considered definitive because of inherent limitations or lack of prospective cohort studies. Secondly, original data were not provided in several of the excluded studies in which only negative results were reported. Thus, effectors such as original correlation coefficients (*r*) and ORs were not provided in the overall analyses. Given this, the power of our conclusion is lower because of missing data. Thirdly, high heterogeneity arose among individual studies related to Spearman correlation between sKlotho and VC or PTH. Although subgroup analysis was conducted, significant heterogeneity persisted in all subgroups in these respects, indicating that this cannot be fully explained by subgroup analysis. Because of the limited number of included studies, we were unable to perform meta-regression to further identify the origin of the heterogeneity. Sensitivity analysis showed that the studies by Zheng et al. and Buiten et al. had substantial effects on heterogeneity. Further examination of these two reports revealed that in the report by Zheng et al., among five studies included, the degree of VC was assessed by computed tomography, and in the report by Buiten et al., among six included studies, the enrolled participants were the oldest among the entire cohort. Differences in baseline features or study design may partly account for heterogeneity. Despite this, the overall results were not changed, as shown with sensitivity analysis, indicating that the results are stable.

## Conclusion

We showed there is an inverse association between sKlotho and VC despite certain limitations, and our conclusion currently supports the potential application of sKlotho as a biomarker for management of VC in CKD patients. However, our results must be interpreted carefully owing to heterogeneity among included publications. To obtain more reliable results, additional studies are required. For example, sKlotho assays should be standardized and sKlotho levels should be measured repeatedly over time and averaged. Importantly, multicenter longitudinal studies or intervention clinical trials with well-designed and controlled confounders are urgently required to validate whether decreased sKlotho predicts increased VC in CKD patients.

## Data Availability Statement

The original contributions presented in the study are included in the article/supplementary material, further inquiries can be directed to the corresponding author/s.

## Author Contributions

QL and LY performed database search, data extraction, and data analysis. QL and SL wrote this manuscript. XY and JY performed study selection and data analysis. JY and SL contributed to the conception and design of the study. All authors reviewed and approved this final version.

## Conflict of Interest

The authors declare that the research was conducted in the absence of any commercial or financial relationships that could be construed as a potential conflict of interest.

## Publisher's Note

All claims expressed in this article are solely those of the authors and do not necessarily represent those of their affiliated organizations, or those of the publisher, the editors and the reviewers. Any product that may be evaluated in this article, or claim that may be made by its manufacturer, is not guaranteed or endorsed by the publisher.
